# Rapid Analysis of Caffeine, Protein and Trigonelline in Ugandan Arabica Coffee Using NIRS and Machine Learning Algorithms

**DOI:** 10.3390/plants15142117

**Published:** 2026-07-09

**Authors:** Joseph Mbihayeimaana, Jimcall Pfumorodze, Ephraim Nuwamanya, Godfrey Sseremba, Vincent Kyaligonza, Paula Iragaba, Michael Kanaabi, James Madzimure

**Affiliations:** 1 School of Law, Africa University, 1 Fairview Road Off Nyanga Road, Mutare P.O. Box 1320, Zimbabwe; mbihajose2008@gmail.com; 2 National Agricultural Research Organization (NARO), Entebbe P.O. Box 295, Uganda; gsseremba16@gmail.com (G.S.); kyalivincent@gmail.com (V.K.);; 3 Department of Law, University of Botswana, Gaborone 0022, Botswana; jimpfumo@gmail.com; 4 College of Agricultural and Environmental Sciences, Makerere University, Kampala P.O. Box 7062, Uganda; nuwamanyaephraim@gmail.com; 5 National Coffee Research Institute (NaCORI), Mukono P.O. Box 185, Uganda; 6 College of Health, Agriculture and Natural Sciences, Africa University, 1 Fairview Road Off Nyanga Road, Mutare P.O. Box 1320, Zimbabwe; madzimurej@africau.edu

**Keywords:** coffee quality, coffee profiling, genetic gain, high-throughput phenotyping, selection, specialty coffee

## Abstract

Coffee is a major export earner for Uganda, raking in over USD 2 billion in 2025. The global price of coffee is tagged to the perceived quality in the cup which in turn is affected by the chemical composition of the green bean. Breeding for market-preferred Arabica coffee varieties is a major objective of coffee breeding programs. Determination of coffee bean chemical constituents is routinely done through expensive, slow and tedious laboratory procedures, making it unsustainable of resource-limited public sector coffee breeding programs. Here, we demonstrate the use of near-infrared spectroscopy (NIRS) and the machine learning algorithms partial least squares (PLS), random forest (RF) and support vector machine (SVM) for the prediction of caffeine, protein and trigonelline in Arabica coffee. NIRS provides a fast, accurate and reliable method of simultaneously predicting multiple sample constituents. Ripe coffee cherries were picked from 172 farmers’ fields, air dried in the laboratory at room temperature and processed to green beans. NIRS spectra were taken on the milled green bean at 400–2500 nm, with a 0.5 nanometer (nm) step. Reference data for caffeine, protein and trigonelline were collected on the same sample scanned with NIRS. A set of 12 spectral pretreatments were applied prior to making calibrations with the PLS, RF and SVM algorithms and 70% of the data as a training set and 30% as a test set. Caffeine content of reference samples ranged from 1.94–3.0 g/100 g, protein content ranged from 11.16–15.94% while trigonelline ranged from 0.94–1.23 g/100 g. The best calibrations for all algorithms and analytes were obtained using raw (untreated) spectra, which gave the same results as the Savitzky–Golay (SG) pretreatment. For caffeine, the best model (R^2^p = 0.89, RMSEP = 0.007, RPD = 3.34) was obtained with the SVM algorithm, while for protein, the best model (R^2^p = 0.98, RMSEP = 0.14, RPD = 6.92) was obtained using the PLS algorithm. Finally, for trigonelline, all three models had very high prediction accuracies (R^2^p = 0.98–0.99, RMSEP = 0.007–0.009, RPD = 8.53–10.52). Collectively, these results demonstrate the potential of using NIRS for rapid and simultaneous prediction of coffee green bean constituents to aid selection decisions.

## 1. Introduction

Coffee is a major export earner for Uganda, attracting over USD 2.4 billion in exports in 2025. Globally, coffee is the most consumed beverage after water with an estimated 400 billion cups sold annually [1]. Coffee processors and consumers have specific quality requirements, which are conferred by the genetics [2,3], environment [4], coffee processing techniques [5] and their brewing conditions [6]. This gives rise to specialty coffee, recognized for its distinctive attributes and associated with significant extra market value [7]. The beverage profile achieved in the cup is linked to the biochemical profile of the green bean [8]. Major biochemical constituents of the green bean include caffeine, chlorogenic acids, trigonelline, proteins, sucrose and trace minerals [9].

Caffeine is an alkaloid of the methylxanthine class and a central nervous system stimulant. It is the most highly consumed psychoactive substance globally [10] with the coffee bean being the primary source of caffeine. Arabica coffee green beans on average contain 0.9–1.5% caffeine on a dry weight basis while Robusta coffee green beans contain 1.2–2.4% caffeine on a dry weight basis [11,12]. Caffeine in the coffee plant is said to be synthesized for protection against insect pests [13]. However, coffee consumers often associate higher caffeine content with “strong coffee”, which may confer a bitter taste [14]. Thus, whereas some markets prefer strong coffee, others demand decaffeinated coffee [15].

Trigonelline is another biochemical that is strongly associated with coffee cup quality [8]. Higher levels of trigonelline are associated with better precursors of flavor. During roasting, trigonelline breaks down into nicotinic acid (vitamin B1) and N-methylpyridinium. These products of degradation of trigonelline are part of the Maillard reaction [16] which affects both aroma and flavor of the coffee beverage, ultimately shaping the sensory profile [8,17].

Green coffee beans also contain appreciable amounts of protein, ~13.5–19.5 g/100 g on a dry weight basis [18]. Moreover, these proteins have a high proportion of amino acids and peptides and thus are potentially of nutritive value [19]. During coffee roasting, the proteins are broken down into amino acids and peptides [20] which react with sugars during the Maillard reaction, thus influencing aroma and flavor [21]. Routine analyses for these biochemicals are hinged upon destructive, slow, laborious and expensive protocols. For example, Kjelder analysis is done for protein analysis while high-performance liquid chromatography (HPLC) is used for caffeine and trigonelline analysis. Given the importance of these biochemicals for coffee quality, it is important that coffee breeders and those involved in quality control have access to tools for fast, accurate and reproducible determination of these constituents in coffee.

A major challenge facing coffee breeding is the long time it takes from variety development to commercial deployment, taking up to 20 years. Thus, the rate of genetic gain on farmers’ fields is slow. For breeding investments to yield high returns, breeders must identify technologies or techniques that can be deployed more effectively. Thus, the deployment of NIRS provides important prospects for increasing future breeding effectiveness and impact for specialty coffees. The lack of tools for rapid and accurate determination of green coffee sample constituents not only limits progress in breeding but also curtails efforts for quality control and rewarding of farmers or producers of premium or specialty coffees based on their biochemical profiles as routine quantification of these in the laboratory is not only slow but is also expensive, requiring deployment of specialized machinery.

Near-infrared spectroscopy (NIRS) provides fast, accurate, reliable and simultaneous predictions of sample constituents with minimal sample preparation [22]. Given its versatility, NIRS has variously been deployed to predict caffeine, trigonelline and 5-caffeoylquinic acid in raw coffee beans using partial least squares (PLS) regression [23], caffeine and protein in Arabica coffee using PLS [24], moisture content in intact green coffee beans [25], chlorogenic acid and caffeine [26] and specialty coffee flavors [27]. Given that data sets are variable, no algorithm is best suited for all data sets. Determination of an appropriate algorithm is rather an ad hoc process [28].

In addition to information on the sample constituents, NIRS spectra may contain noise such as stray light and sample background [29]. This noise can be corrected by the application of mathematical pretreatments. An important step in developing NIRS calibrations is determining whether preprocessing is necessary and which preprocessing method to use. Savitzky–Golay (SG) smoothing, derivative, baseline correction, standard normal variate (SNV) and combinations of these methods have previously been used for NIRS calibrations [30,31]. Derivative preprocessing helps to eliminate baseline offset and drift. It also helps to overcome spectral band overlap [32]. Baseline correction spectral pretreatments eliminate spectral baseline drift by artificially pulling the spectrum to a zero baseline [33]. Standard normal variate (SNV) on the other hand reduces spectral distortion due to dispersion [34].

This study therefore aimed to evaluate the prediction accuracy of NIRS for caffeine, proteins and trigonelline in green Arabica coffee beans using the algorithms partial least squares (PLS), random forest (RF) and support vector machine (SVM). This is the first attempt to use NIRS to predict Ugandan Arabica coffee green bean sample constituents.

## 2. Results

### 2.1. Variability of Caffeine, Protein and Trigonelline in the Green Coffee Bean Samples

**Figure 1:**
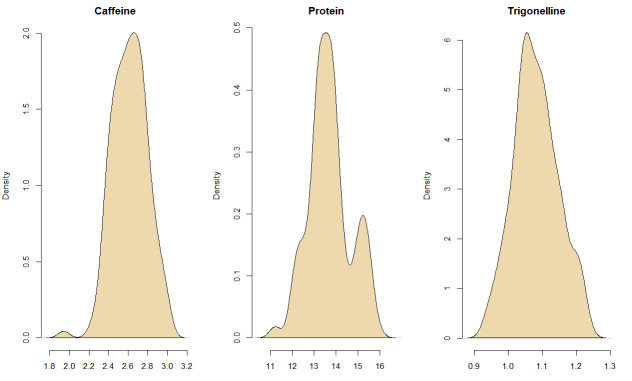
Variability of caffeine, protein and trigonelline among the reference Arabica coffee green bean samples collected from farmers’ fields in Uganda.

The levels of caffeine in the samples ranged from 1.94 g/100 g to 3.00 g/100 g with a mean of 2.62 g/100 g and a median of 2.62 g/100 g. On the other hand, protein content ranged from 11.16% to 15.94% with a mean of 13.70% and a median of 13.00% while trigonelline content ranged from 0.94 g/100 g to 1.23 g/100 g with a mean of 1.08 g/100 g and a median of 1.08 g/100 g. Summary statistics of the reference data are provided Table 1. The reference data for caffeine, protein and trigonelline was diverse, thus suitable for model development (Figure 1).

### 2.2. NIRS Spectra and Spectral Pretreatments

**Figure 2:**
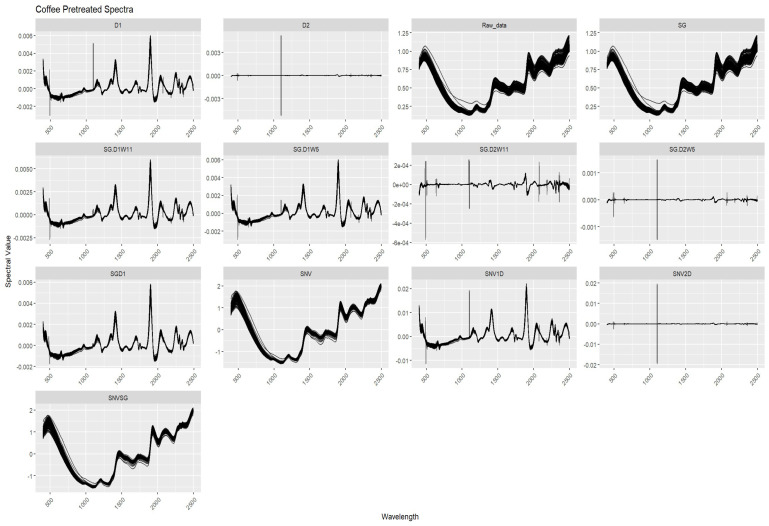
Plot of raw (untreated) spectra and treated spectra showing impact of spectral pretreatments on the spectral patterns.

Spectral data quality control was conducted to identify outliers. There were no spectra flagged as outliers. Spectral pretreatments were therefore applied to all 172 spectra. We observed peaks around 500 nm, 1450–1500 nm, 1600–1700 nm, 1900–1950 nm, 2050–2100 nm and 2300–2400 nm (Figure 2). The peak in the visible region (500 nm) is associated with colored compounds, probably due to the color of the green coffee beans. The 1450–1490 nm region is associated with the first overtone of the NH stretch. The region 1920–1980 nm is associated with the combination of NH stretch and deformation [35], the 2050–2100 nm is region is associated with the combination of CH stretch and NH bend [36], the 2180–2240 nm region is associated with the combination of CH3 stretch and deformation while the 2260–2340 nm region is associated with the combination of C-H and C-N vibrations [37].

The peaks observed at ~1680–1720 nm, associated with the aromatic C-H stretch and first overtone, and the combination region ~2050–2100 nm are associated with caffeine. Similarly, the peak at ~2300–2400 nm which is the second overtone (C=O stretch) is also associated with caffeine but has a high likelihood of overlapping with other carbohydrate signals like cellulose and hemicellulose or lipids [38].

Trigonelline is an alkaloid with distinct NIRS features given its distinct N-H combination bands which are less common in other coffee constituents. Trigonelline synthesis is carried out by enzymatic methylation of nicotinic acid [39]. Its characteristic N-H and C-H vibrations give rise to strong overtone and combination bands associated with specific spectral regions, 1450–1490 nm [39,40]. Proteins on the other hand have characteristic amide (N-H) and C-H bonds and therefore have distinct absorption bands in the region ~1490–1580 nm, which is the first overtone of N-H stretch and the primary region associated with protein variability. The region ~2050–2100 is associated with the N-H stretch and amide II combination, the ~2170–2240 nm region is associated with the N-H and amide III combination, the ~2260–2360 nm region is associated with C-H stretch combination bands (CH2, CH3) from amino acid side chains, and the ~2300–2340 nm region is associated with the second overtone of C=O stretch (amide I) [36,41].

### 2.3. Effect of Spectral Pretreatments on Prediction Accuracy of PLS, RF and SVM Algorithms for Caffeine

Using the PLS algorithm, the highest squared Pearson’s correlation between predicted and observed test set values (R^2^p = 0.855) was obtained using raw (untreated spectra) and SG-treated spectra and it was significantly higher (*p* < 0.001) than those of other pretreatments. These had low root mean squared error of prediction (RMSEp = 0.09) and low bias (0.032). The coefficient of multiple determination of cross-validation (R^2^cv) was 0.554, the root mean squared error of cross-validation (RMSECV) was 0.254, the ratio of performance to interquartile difference (RPIQ) was 2.964, and the concordance correlation coefficient (CCC) was 0.909. The root mean squared error of prediction (RMSEp) was 0.09 while the ratio of standard deviation of observed test set values to RMSEp (RPD) was 2.946. Finally, the squared Spearman’s rank correlation between predicted and observed test set values (R^2^sp) was 0.874. Whereas raw data and SG-pretreated spectra did not differ in any metric, they were significantly different from other pretreatments (Table 2).

Similar to PLS, using the SVM algorithm, the highest R^2^p (0.894) was obtained using raw (untreated spectra) and SG-treated spectra followed by SNV- and SNVSG-pretreated ones. Whereas these were not significantly different from each other for all metrics at α = 0.05, they differed significantly (*p* < 0.001) from other pretreatments for all other metrics with the exception of bias (Table 3).

A similar but slightly different pattern of performance was observed for the random forest algorithm where the highest R^2^p (0.834) was obtained using raw (untreated spectra), followed by SG-treated spectra (R^2^p = 0.826). These had low RMSEp (0.094, 0.095) and low bias (0.015, 0.031) for raw and SG-treated spectra respectively. The RPIQ was (3.992, 2.743) the concordance correlation coefficient (CCC) was (0.939, 0.89), the RPD was (3.342, 2.312), while the squared Pearson correlation between predicted and observed test set values was (0.92, 0.831) for raw and SG-treated spectra respectively (Table 4).

**Figure 3:**
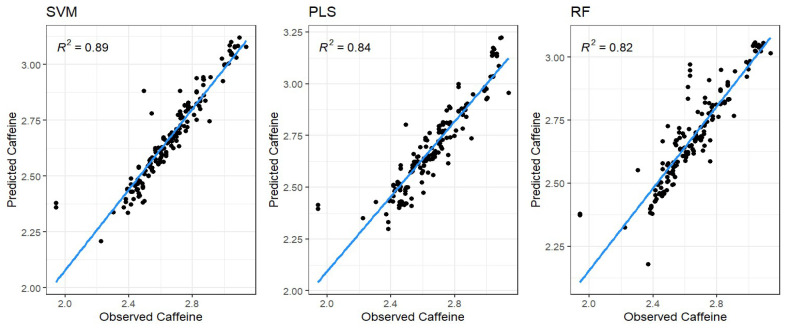
Scatter Plots of Observed versus Predicted values for caffeine using Random Forest (RF), Support Vector Machine (SVM) and Partial Least Squares (PLS) algorithms with raw spectra.

Overall, applying a pretreatment rather than SG significantly reduced the prediction accuracy of the PLS, SVM and RF algorithms for caffeine. The highest prediction accuracy (R^2^p = 0.89) was achieved with the SVM model, followed by PLS (R^2^p = 0.84) and RF (R^2^p = 0.82) (Figure 3). The bias was not significantly different across spectral pretreatments for all models at a 5% alpha level. This suggests the spectral preprocessing is robust and any improvements in model accuracy are likely to be obtained either by improving the quality and representativeness of the reference data or by employing different modeling techniques like stacked ensembles which have been shown to improve NIRS prediction accuracy [42,43,44,45].

Our results are comparable to [26] who evaluated the potential of NIRS combined with SVM and PLS for prediction of caffeine in intact coffee beans, reporting accuracies of R^2^p = 0.75, RPD = 1.94 with PLS and R^2^p = 0.94, RPD = 4.38 with SVM. However, ref. [24] reported better performance with the PLS algorithm (R^2^p = 0.989, RPD = 19.943). When predicting caffeine content of green coffee beans, the choice of algorithm is important as it impacts on accuracy of the predictions. NIRS combined with SVM can be deployed in routine quality control of coffee based on defined thresholds of acceptability [46].

### 2.4. Effect of Spectral Pretreatments on Prediction Accuracy of PLS, RF and SVM Algorithms for Protein

In general, prediction accuracies for the three algorithms were high for protein. Using PLS, the R^2^p and RMSEp values ranged from (0.79, 0.45) using SNV2D pretreatment to (0.98, 0.14) using SG pretreatment and raw spectra respectively (Table 4). The R^2^cv values were all high for the different spectral pretreatments (0.95–0.97) and the bias values were all negative. The RPD ranged from 2.19 with SNV2D pretreatment to 6.92 for SG pretreatment and raw spectra. The CCC values were all high, ranging from 0.88 to 0.99. The squared Spearman’s rank correlation between predicted and observed test set values ranged from 0.77 with SNV2D pretreatment to 0.96 with raw and SG-pretreated spectra (Table 5).

Using the SVM algorithm, we observed a similar trend to the PLS algorithm in performance. The squared Pearson’s correlation between predicted and observed test set values ranged from 0.83 with SNV2D to 0.97 with SG-pretreated and raw spectra. The RMSEp values ranged from 0.4 for SNV2D to 0.17 for SG. Bias values were negative, ranging from −0.014 (SNV2D) to −0.016 (SG and raw spectra) with corresponding CCC values of 0.92 (SNVD2) and 0.98. The RPD ranged from 2.56 to 5.772 while RPIQ ranged from 0.9 to 0.98. The R^2^sp values ranged from 0.86 (SNV2D) to 0.95 (SG-treated and raw spectra) (Table 6).

The trend of performance observed for PLS and SVM was reproduced with the RF algorithm. The R^2^p values were high, ranging from 0.78 (SNV2D) to 0.97 (SG and raw data). The corresponding RMSEp values were 0.46 (SNV2D) and 0.18 for SG-pretreated and raw spectra respectively. The ratio of performance to deviation ranged from 2.1 (SNV2D) to 5.4 (SG and raw spectra) with corresponding CCC values of 0.9 and 0.98. The values of bias were negative, ranging from −0.016 (SN2D) to −0.004 (SG and raw spectra) with corresponding R^2^sp values of 0.72 (SG2D) and 0.96 (SG and raw spectra) (Table 7).

**Figure 4:**
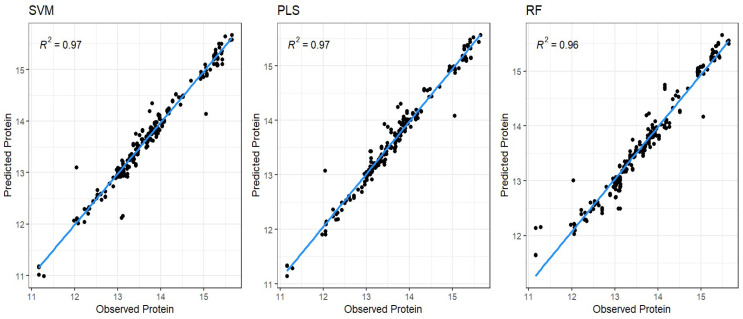
Scatter Plots of Observed versus Predicted values for protein using Random Forest (RF), Support Vector Machine (SVM) and Partial Least Squares (PLS) algorithms with raw spectra.

Overall, prediction accuracies for protein were high, regardless of the model used and whether or not spectral pretreatments were applied. Similar to the PLS and SVM algorithms, the highest prediction accuracies were obtained using either SG-pretreated spectra or raw spectra with similar R^2^p values being reported by all three algorithms (Figure 4). These results are comparable to [24] who reported high prediction accuracy for protein in Arabica coffee beans using NRS and PLS (R^2^p = 0.989, RPD = 11.896). These high prediction accuracies can be attributed to the fact that proteins have distinct amide (N-H) and C-H absorption bands [36,41], making NIRS a reliable tool for quality control based on preset thresholds.

### 2.5. Effect of Spectral Pretreatments on Prediction Accuracy of PLS, RF and SVM Algorithms for Trigonelline

The PLS algorithm had high prediction accuracies, which varied according to the spectral pretreatment applied. The lowest accuracy (R^2^p = 0.76, RMSEcv = 0.025, RMSEp = 0.034, bias = 0.002) was recorded with the SNV2D pretreatment while the highest accuracy (R^2^p = 0.99, RMSEcv = 0.01, RMSEp = 0.007, bias = 0.001) was recorded with raw spectra and SG pretreatment. The RPD ranged from 2.08 (SNV2D) to 10.53 (SG, raw data) with corresponding CCC values of 0.84 and 0.99. The R^2^sp values ranged from 0.73 (SNV2D) to 0.98 (SG, raw spectra) (Table 8).

Similar to the PLS algorithm, prediction accuracy varied with the spectral pretreatment applied. The lowest accuracy (R^2^p = 0.896, RMSEp = 0.022, bias = 0.002, RPD = 3.25) was observed with SGD1 pretreatment while the most accurate predictions (R^2^p = 0.97, RMSEp = 0.008, bias = 0.001, RPD = 9.1) were made with raw spectra and/or SG-pretreated spectra. The squared Spearman’s rank correlation between predicted and observed test set values ranged from 0.86 (SGD1) to 0.98 (SG-pretreated, raw spectra) (Table 9).

Using the RF algorithm, model accuracy was influenced by the spectra pretreatment applied. However, in general, the prediction accuracies were high. The lowest prediction accuracy (R^2^p = 0.74, RMSEp = 0.036, bias = 0.003, RPD = 1.9) was posted by the SNV2D pretreatment while the most accurate predictions (R^2^p = 0.98, RMSEp = 0.009, bias = 0.001, RPD = 8.53)) were observed with the SG pretreatment and raw spectra. The CCC ranged from 0.79 (SNV2d) to 0.99 (raw spectra). The squared Spearman’s rank correlation between predicted and observed test set values ranged from 0.71 (SNV2D) to 0.98 (SG-pretreated, raw spectra) (Table 10).

**Figure 5:**
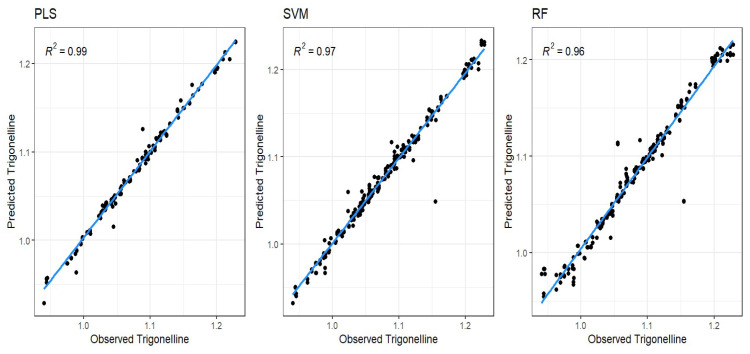
Scatter Plots of Observed versus Predicted values for Trigonelline using Partial Least Squares (PLS), Support Vector Machine (SVM), and Random Forest (RF) algorithms with raw spectra.

Similar to protein content predictions, the prediction accuracies for trigonelline were generally high, regardless of model used and whether or not spectral pretreatments were applied. The highest prediction accuracies were obtained using either SG-pretreated spectra or raw spectra with a narrow range of variation of R^2^p values (0.96–0.99) between the models (Figure 5). The performance of the PLS model for trigonelline reported in our study is superior to that reported by [40] from intact coffee beans (r = 0.98, RPD = 2.98) and roasted coffee beans (r = 0.95–0.99) [47] given the strong generalizing ability of our PLS model combined with raw spectra (RPD = 10.53). NIRS can be readily used to select for coffee quality based on an acceptable cut-off or preset threshold for trigonelline.

**Figure 6:**
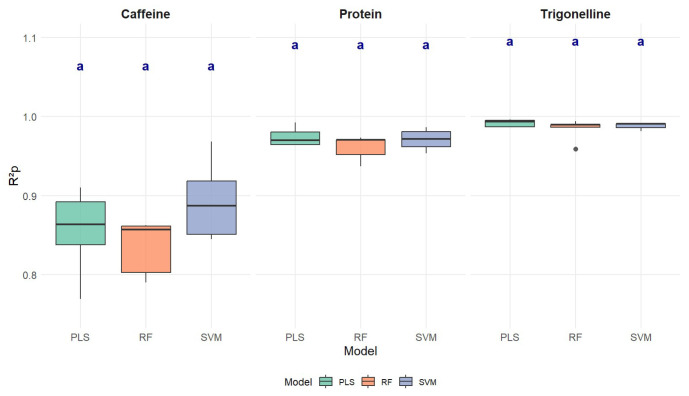
Box plot showing variability in coefficient of determination for the prediction set (R^2^p) for the different analytes and prediction models using raw spectra. PLS = Partial Least Squares, RF = Random Forest, SVM = Support Vector Machine. The letter a represents results of the Kruskal-Wallis test indicating no signficant difference among the alogorithms in prediction accuracy for caffeine, protein and trigonelline.

Overall, based on raw spectra the PLS and SVM models (R^2^p = 0.97) gave the best predictions for protein, which had small variability over the 10 iterations (Figure 6). For caffeine, the best predictions (R^2^p = 0.89) were given by the SVM model but variability in prediction accuracy was high across the 10 iterations. Finally, for trigonelline, the best prediction accuracy was obtained with PLS (R^2^p = 0.99) with small variability in prediction accuracy for PLS and SVM, but with an outlier prediction for RF. However, in the grand scheme of things, there was no statistically significant difference (α = 0.05) among the models in their prediction accuracy for a particular analyte given that they were assigned the same letter (a) when the Kruskal–Wallis test was done (Figure 6). Summary statistics for the different models across the 10 iterations are presented in Table S1.

### 2.6. Influential Wavelengths for Caffeine, Protein and Trigonelline Prediction

**Figure 7:**
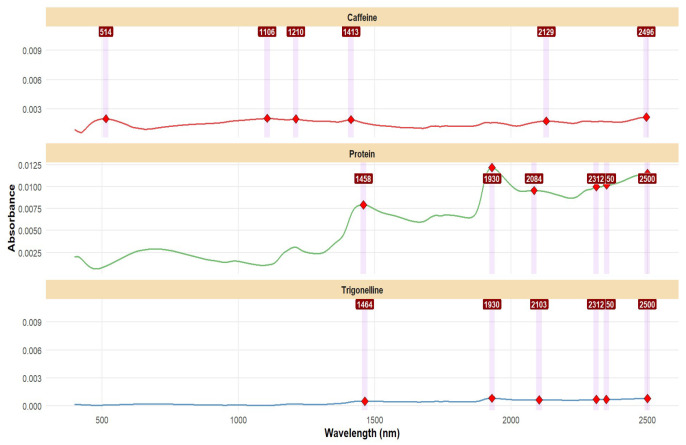
Influential spectral wavelengths for caffeine, protein and trigonelline content prediction in green Arabica coffee beans.

Using the PLS model, a plot of absorbance against wavelength for the different analytes revealed peaks at different wavelengths which correspond to key or influential wavelengths for predicting the analyte. For caffeine prediction, we observed peaks at 514 nm, 1106 nm, 1210 nm, 1432 nm, 2129 nm, and 2496 nm (Figure 7). For protein, we observed prominent peaks at 1498 nm, 1930 nm, 2084 nm, 2312 nm, 2350 nm and 2500 nm, while for trigonelline, we observed peak absorbances at 1464 nm, 1930 nm, 2103 nm, 2313 nm, 2350 nm and 2500 nm (Figure 7). The absorbance peaks for protein and trigonelline had a striking similarity but differed in the 1700–2050 region, where protein is seen to peak earlier (1458 nm) than trigonelline (1464 nm). Whereas the trigonelline peaks typically correspond to N-H stretching and C-H and N-H combination regions, the protein peaks correspond to amide (N-H) bonds and amino acid side chains. The variable importance analysis provided spectral interpretation that aligns with known absorption bands for caffeine, trigonelline and proteins [35,36,37,38,40,41]. As demonstrated in a previous study, identification of influential wavelengths does not only reduce the computational resources required for model development but could also potentially improve NIR prediction model accuracy [31].

## 3. General Discussion

The impressive model performances achieved using untreated spectra can be attributed to our elaborate sample preparation protocol which involved milling the green beans to create a uniform sample as opposed to whole green beans which are likely prone to light scatter and thus noisy spectra [48]. Overall, prediction accuracies differed for caffeine, protein and trigonelline, with trigonelline registering the highest accuracies, closely followed by protein, with caffeine accuracies being distant, albeit high. These differences can be attributed to the fact that NIRS detects vibrational overtones of O-H, N-H and C-H bonds and the accuracy of its predictions is dependent on how distinct and strong these vibrational overtones are in the sample matrix [49]. Trigonelline (C_7_H_7_NO_2_) has prominent N-H bonds which generate strong, well-resolved absorption peaks and thus high prediction accuracies [39]. Similarly, proteins have long-chain amino acids containing N-H, C=O and C-H bonds, thus yielding robust signals that can be associated with the high accuracies obtained in the study [50]. Similarly, lower prediction accuracies for caffeine could be attributed to its chemical structure, which is dominated by weak and overlapping C-H, C=O and C-N bonds [51], which ultimately produce a weak signal to noise ratio. The finding that raw spectra consistently outperformed treated spectra (with the exception of SG-treated spectra which yielded identical performance) is key in future efforts to deploy NIRS for rapid phenotyping of breeding populations and coffee quality control. The implication is that the spectra collection protocol has already been optimized, eliminating noise and thus relegating the need for the computational-resource-intensive spectral pretreatments. The SG pretreatment had near-identical performance to the raw spectra. The SG filter functions to improve NIRS predictions by smoothing to reduce random noise in the spectra [52]. Because no outliers were detected in the spectra, the SG filter had minimal effect on the spectra, thus producing its sharp resemblance to the untreated spectra both in the spectral plots and in prediction performance with the different algorithms. Pretreatments that lowered the prediction accuracy of models likely disrupted the pattern in spectra, introducing noise and ultimately increasing the noise:signal ratio. A similar trend has been reported for binary classification of low- and high-cyanogenic cassava using NIRS and machine learning algorithms [31]. Similarly, Braun et al. [52] demonstrated while working on soybean, maize and triticale that no single spectral pretreatment was a perfect fit for all data sets. The algorithms did not differ much in their prediction accuracies for the same trait but generally yielded models with high accuracy and high RPD. The choice of algorithm has a high impact on the computational resources required to develop prediction equations. PLS being less computationally demanding compared to RF and SVM makes it a model of choice in situations where model performance is comparable.

The RPD (3.34) obtained for caffeine with the SVM algorithm implies that the model is suitable for screening breeding populations [41] while the high RPDs (6.92) obtained for protein with PLS and trigonelline with all models (>8.0) imply that these models can reliably predict samples outside the test samples and are suitable for most purposes, including quality control [36,53]. NIRS technology is not only applicable for screening but is also useful for flavor profiling [4], supporting applications for coffee geographical indication [54]. Given that caffeine, protein and trigonelline are key determinants of end-user coffee cup quality, the prediction equations developed can be readily deployed for quality control purposes during coffee processing to eliminate coffee that does not meet quality requirements based on its biochemical profile and the predicted quality of the final cup. Furthermore, the procedure can be deployed to authenticate geographical origin of coffee in situations where coffee biochemical profiles have already been matched to geographical locations. Indeed, NIRS technology has been variously deployed for authentication of geographical origin of coffee [54,55,56], verification of geographical indication [57] and prediction of specialty coffee flavors [27,53]. In green coffee beans, NIRS has been used to instantly predict various biochemical constituents, including moisture content, chlorogenic acid, caffeine, total sugar, sucrose and phenolic compounds [58,59,60]. NIRS has found similar applications in apples [61] cuttlefish [62] and millet [63]. The high prediction accuracies attained for caffeine, trigonelline and protein imply that NIRS can be used to predict specialty Arabica coffee flavors by simultaneously analyzing these constituents. Moreover, the application of NIRS in selection is essential in modernization of coffee breeding programs, as envisioned by Kawuki et al. [64], and will be key in development of coffee varieties with desirable quality characteristics as it will allow for rapid and cost-effective phenotyping of large early-stage trials. The success of NIRS technology in predicting the constituents of roasted coffee beans [47,65,66,67] paints a highly promising picture for the industry to adopt the technology for monitoring roasting processing, a key step in production of high cup quality. A major limitation of the study is that the sample number (n = 172) was too small to represent the entire Arabica diversity in farmers’ fields in Uganda, although there was diversity for caffeine, protein and trigonelline in the samples. However, the sample collection targeted major Arabica coffee growing regions of Uganda, in two distinct highland agroecological zones which are characterized by differences in altitude, weather, soils and probably Arabica coffee genotypes cultivated as no distinct Arabica coffee varieties have been released by Uganda’s coffee breeding program. So, whereas the geographical area covered was large, only a few samples were picked. This likely reduced the overall usefulness of the models as a large portion of the natural diversity of these analytes was not captured. Moreover, only cross-validation was done during calibration development. The lack of an external validation set leaves room for doubt on the generalizability of the models. For better generalizability, future studies could consider collecting more samples from more farmers spread across diverse geographical locations and including an external validation set to test robustness of the models. Because the external validation set is not involved in development of the predictions, it is a good standard to test the extent to which the developed model can predict analytes in real-world situations. Without an external validation set, the applicability of these results is limited to the study material. The lack of reference data and therefore calibrations for moisture content and chlorogenic acid content of the green coffee beans was a major oversight that subsequent work should address for these are essential in determining the resultant quality of the roasted bean [68,69,70,71]. The use of the full spectral wavelength and multiple spectral pretreatments consumed considerable computing resources. In future, the use of specific wavelengths associated with analytes under study could be considered.

## 4. Materials and Methods

### 4.1. The Study Area and Sample Collection

**Figure 8:**
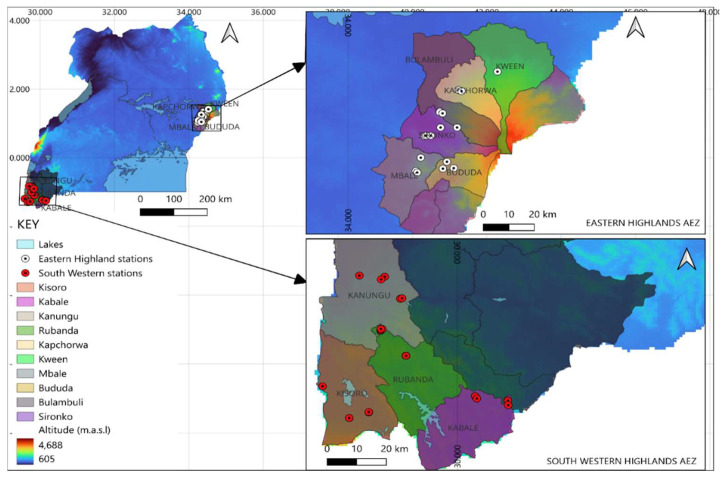
Map of Uganda showing the geographical locations from which Arabica coffee samples were collected in the eastern highland and southwestern highland agroecological zones.

The study was conducted in the southwestern highland agroecological zone of Uganda comprising the districts of Kisoro, Rubanda, Kabale, and Kanungu and in the eastern highland agroecological zone of Uganda comprising of Mbale, Kapchorwa, Kween, Bulambuli, Sironko, and Bududa districts (Figure 8). The southwestern highland agroecological zone is characterized by an altitude of 1260 to over 2800 m above sea level, with annual daily temperatures of 13–23 °C and bimodal rainfall patterns at 1000–1500 mm per annum. The soils are variable across the highland ranges. The environment comprises thick natural forests, the Muhabura mountain range and crater lakes. The eastern highland agroecological zone on the other hand is characterized by altitude of 1100 to over 2800 m above sea level, a bimodal rainfall pattern with 1000–1500 mm rain per annum, volcanic fertile soils and daily temperatures of 15–30 °C.

### 4.2. Coffee Sampling and Sample Collection

Coffee bean samples were collected from eastern highland agroecological zone and southwestern agroecological zone of Uganda using a stratified composite approach. For each zone, 3 districts were covered. At each farm, 5 trees bearing ripe cherries were randomly selected along a diagonal and ripe cherries picked. Collectively, about 800 g of ripe cherries were taken and homogenized per farm. All samples were labeled, sealed in paper bags and transported in open boxes to allow for drying and moisture loss during transit. In the laboratory, the sample bags were put in open spaces with proper aeration and dried to 13% moisture content at room temperature. The dried samples were then stored at 4 °C and protected from light.

### 4.3. Collection of Reference Data for Protein, Trigonelline and Caffeine

#### 4.3.1. Protein Determination by Kjeldahl Analysis

Protein analysis was done following the Kjeldahl protocol [72]. Briefly, total nitrogen was measured and converted to crude protein using a nitrogen-to-protein conversion factor for coffee powders which is 5.30–5.40 for coffee-specific amino acid profiles. The reagents used included concentrated H_2_SO_4_, catalytic tablets (K_2_SO_4_ + Cu), NaOH (40–50%), boric acid (4%), standardized HCl titrant and mixed indicator. Procedurally, 0.5–1.0 g sample was weighed into a Kjeldahl flask followed by addition of 5 g catalyst and 15 mL H_2_SO_4_. The solution was digested at ~420 °C until it was clear after 75 min. The solution was cooled followed by addition of excess NaOH. The solution was then steam-distilled for ammonia into a boric acid receiver. The resultant solution was titrated into ammonium borate with standard acid to the endpoint. For protein determination, the percentage of nitrogen was calculated and converted to protein: %Protein = %N × 5.35. The results were corrected on a dry basis using measured moisture. For quality control, a reagent blank was prepared for every batch of analysis while triplicate sample analyses were also carried out. It was also ensured that digestion completeness was achieved after observation of a clear green solution.

#### 4.3.2. Trigonelline Determination (HPLC-UV, Aqueous Extraction)

Trigonelline was determined using HPLC aqueous extraction procedures with UV detection using a reversed-phase C18 column with a highly aqueous, acidic mobile phase [73,74]. The reagents used included trigonelline hydrochloride analytical standard (≥98%), ultrapure water, LC-MS grade acetonitrile (ACN) and formic acid. Procedurally, 0.50 g of coffee powder was weighed into a 50 mL tube. To this solution 25.0 mL water: ACN (20:80, *v*/*v*) was added followed by 0.1% formic acid. The resultant solution was vortexed for 1 min followed by sonication for 15 min at room temperature. The solution was finally centrifuged for 10 min at ≥4000 g. The supernatant was filtered through 0.22 µm PTFE or C18 into HPLC vials. For determination, a reversed-phase column, C18, 150 × 4.6 mm, 5 µm, was used. The mobile phase consisted of 0.1% formic acid in water (A): ACN (B) while isocratic 95:5 (A: B) over 10 min was used. Detection was carried out at 265–268 nm for external calibration with 6–8 levels (e.g., 0.5–100 mg L^−1^) in extraction matrix (matrix-matched). Under the described conditions, the limit of detection (LOD) and limit of quantification (LOQ) for trigonelline were determined based on a signal to noise ratio of 3:1 and 10:1 respectively; LOD: 0.15 mg L^−1^ and LOQ: 0.50 mg L^−1^. External matrix-matched calibration was performed using 6–8 concentration levels (e.g., 0.5–100 mg L^−1^) of trigonelline standard prepared in the extraction matrix. All determinations were performed in duplicate.

#### 4.3.3. Caffeine Determination (HPLC-UV, Aqueous–Organic Extraction)

Caffeine was determined using HPLC aqueous extraction procedures with UV detection using a reversed-phase C18 column [75]. The reagents used included caffeine analytical standard (≥99%), HPLC grade water, methanol and 0.1% aqueous formic acid. For extraction, 0.25 g of coffee powder was weighed into a Falcon tube. To the powder, 25.0 mL of water:methanol (50:50) with 0.1% formic acid was added. The solution was vortexed for 2–4 min and later sonicated for 15 min at room temperature followed by centrifugation for 10 min at ≥4000 g. The resultant solution was filtered using a 0.22 µm filter. For determination, a C18 column, 150 × 4.6 mm, 5 µm (or 100 × 2.1 mm, 3 µm), was used. The mobile phase consisted of A = 0.1% formic acid in water; B = ACN. The isocratic phase was 85:15 (A:B) at 1.0 mL min^−1^, 30 °C, in 5–10 µL while detection was carried out at 272 nm. For calibration and standardization an external caffeine standard with 6–8 levels (20–200 mg L^−1^), matrix-matched, was used. Caffeine reference data were collected on only 155 samples which were used for downstream analyses. The determination was undertaken in duplicate. Details of the HPLC parameters set are given in Table 11. The HPLC chromatograms are given in Supplementary Figure S1. Data quality parameters for HPLC-generated reference data are given in Table S2.

#### 4.3.4. NIRS Spectra Collection

The dried samples were then ground to powder and sieved using a 250 µm sieve mesh. NIRS spectra were collected from the ground sample using a filled small sample cup of the FOSS NIRS^TM^ (Model DS2500, Serial No. 91793020). The spectra were recorded from 400 nm to 2500 nm at 0.5 nm intervals and saved as the average of 32 scans per sample. Two spectra were collected per sample. Downstream analyses were conducted on the aggregated spectra. In total, NIRS spectra were collected from 172 samples.

### 4.4. Data Analysis

#### 4.4.1. NIRS Spectral Data Analysis

The *waves* R package (version 0.2.6) [76] was used to visualize the spectra and identify outliers based on Mahalanobis distance using the *plot_spectra* function, apply spectral pretreatments using the *pretreat_spectra* function and to run calibrations using the *test_spectra* function. To detect outliers, the calculated Mahalanobis distance was compared against a chi square distribution with degrees of freedom equal to the number of spectral data columns and an alpha level of 0.05. Biplots of observed versus predicted values were generated using the ggplot2 R package [77].

#### 4.4.2. Spectral Pretreatments

After checking spectra for outliers, spectral pretreatments were applied as follows: 1 = Raw data (untreated spectra), 2 = Standard normal variate (SNV), 3 = Standard normal variate and first derivative (SNVD1), 4 = Standard normal variate and second derivative (SNVD2), 5 = First derivative (D1), 6 = Second derivative (D2), 7 = Savitzky–Golay filter (SG), 8 = Standard normal variate and Savitzky–Golay filter (SNVSG), 9 = Gap-segment derivative, window size = 11 (SGD1), 10 = Savitzky–Golay filter and first derivative, window size = 5 (SGD1W5), 11 = Savitzky–Golay filter and first derivative, window size = 11 (SGD1W11), 12 = Savitzky–Golay filter and second derivative, window size = 5 (SGD2W5), 13 = Savitzky–Golay filter and second derivative, window size = 11 (SGD2W11). The role of each spectral pretreatment in correcting noise in spectra is elaborated in Supplementary Table S3.

#### 4.4.3. Calibration Development

Prior to calibration development, the laboratory reference data was merged with the NIRS spectra. For all the algorithms, cross-validation was performed using stratified sampling where 70% of the data was used as a training set while the remaining 30% was used as a test set [22,78], using the wrapper function *test_spectra()* in the waves R package, version 0.2.6. Spectra were aggregated using the *aggregate_spectra( )* function, which returned a mean absorbance at each wavelength per sample by setting the *agg.function* call to mean. Model development was performed in 10 iterations. In each iteration, the *train.proportion()* function was used to randomly split the data into a 70% training set and 30% test set for model evaluation, thus generating 10 models per algorithm. The final model performance statistics were the mean of the 10 model iterations. Overall, models were run on 172 samples and 4201 predictors for protein and trigonelline while only 155 samples were run for caffeine. The metric used to determine the best model was the coefficient of determination (“Rsquared”). For partial least squares regression, we fitted the kernel algorithm with tune length set to 50. Laboratory data was set as a reference and the search space for tuning the PLS hyperparameter *ncomp* was set to 50.

**Figure 9:**
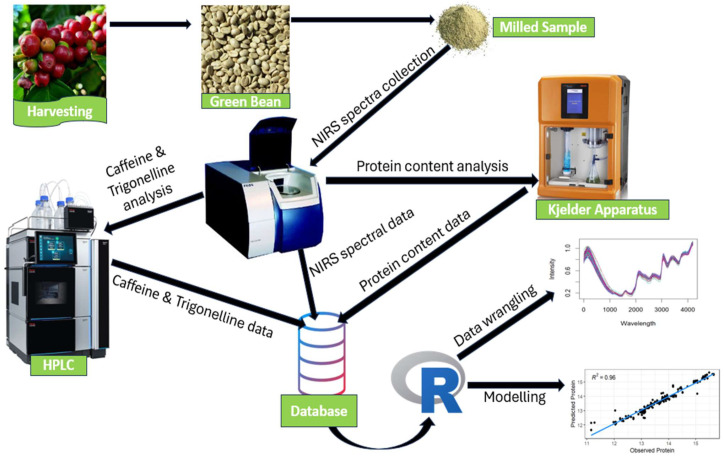
Workflow showing sequence of events from harvesting of ripe Arabica coffee cherries to the development of NIRS prediction models for caffeine, protein and trigonelline.

The support vector machine algorithm was run with a linear kernel, following a five-fold cross-validation, with the tuning parameter *C* set to 1, which is the default value for the waves R package. On the other hand, random forest regression was done using 500 trees. To test for significance of observed differences in model performance metrics following different spectral pretreatments, we conducted analysis of variance (ANOVA) using the *aov* function and separated means of the 10 runs using the *HSD.test* function of the agricolae R package [79]. For PLS models, variable importance results were extracted across iterations to determine the wavelengths with the greatest influence for predicting the different analytes. Plots of absorbance at different wavelengths were generated to visualize the peaks associated with the different analytes. A summary of the workflow from sample collection to prediction model development is presented in Figure 9.

## 5. Conclusions

The results presented in this paper strongly suggest that NIRS combined with machine learning algorithms has immense potential for the simultaneous prediction of caffeine, protein, and trigonelline in Arabica coffee. The observation that raw spectra performed as well as SG-pretreated spectra and better than all other pretreatments eases the data analysis pipeline, cognizant of the elaborate sample preparation involving milling green beans. Thus, NIRS combined with machine learning algorithms can be reliably applied to predict sample constituents without elaborate spectral pretreatments.

The development of high-performance models, particularly the SVM model for caffeine, the PLS model for protein, and the generally strong predictions for trigonelline, suggests that NIRS has strong potential as a rapid screening, selection and quality control tool for coffee.

## Figures and Tables

**Table 1:** Summary statistics for the caffeine, protein and trigonelline reference data.

	Min	1st Quartile	Median	Mean	3rd Quartile	Max	Range
Caffeine (g/100 g)	1.942	2.488	2.617	2.616	2.739	3.00	1.058
Protein (%)	11.160	13.120	13.660	13.700	14.160	15.940	4.780
Trigonelline (g/100 g)	0.941	1.041	1.082	1.082	1.123	1.228	0.287

**Table 2:** Mean performance statistics of NIRS for predicting composition of Caffeine in green Arabica coffee beans using the PLS algorithm.

Pretreatment	RMSEp	R^2^p	RPD	RPIQ	CCC	Bias	RMSEcv	R^2^cv	R^2^sp
Raw_data	0.090 ^b^	0.855 ^a^	2.457 ^a^	2.946 ^a^	0.909 ^a^	0.032 ^a^	0.254 ^c^	0.554 ^a^	0.874 ^a^
SG	0.089 ^b^	0.855 ^a^	2.457 ^a^	2.946 ^a^	0.909 ^a^	0.032 ^a^	0.254 ^c^	0.554 ^a^	0.874 ^a^
SNV	0.145 ^a^	0.600 ^b^	1.499 ^b^	1.765 ^b^	0.761 ^b^	0.032 ^a^	0.302 ^ab^	0.367 ^bc^	0.582 ^b^
SNV1D	0.165 ^a^	0.555 ^b^	1.323 ^b^	1.552 ^b^	0.721 ^b^	0.038 ^a^	0.278 ^bc^	0.460 ^ab^	0.591 ^b^
SNV2D	0.202 ^a^	0.312 ^b^	1.070 ^b^	1.263 ^b^	0.528 ^b^	0.049 ^a^	0.302 ^ab^	0.363 ^bc^	0.303 ^b^
D1	0.169 ^a^	0.545 ^b^	1.284 ^b^	1.511 ^b^	0.716 ^b^	0.032 ^a^	0.294 ^abc^	0.401 ^abc^	0.591 ^b^
D2	0.202 ^a^	0.316 ^b^	1.078 ^b^	1.270 ^b^	0.481 ^b^	0.037 ^a^	0.321 ^a^	0.274 ^c^	0.300 ^b^
SNVSG	0.145 ^a^	0.602 ^b^	1.493 ^b^	1.758 ^b^	0.761 ^b^	0.033 ^a^	0.254 ^ab^	0.367 ^bc^	0.588 ^b^
SGD1	0.159 ^a^	0.570 ^b^	1.391 ^b^	1.635 ^b^	0.735 ^b^	0.033 ^a^	0.295 ^abc^	0.396 ^abc^	0.587 ^b^
SG.D1W5	0.169 ^a^	0.547 ^b^	1.289 ^b^	1.517 ^b^	0.717 ^b^	0.032 ^a^	0.294 ^abc^	0.400 ^abc^	0.587 ^b^
SG.D1W11	0.165 ^a^	0.557 ^b^	1.317 ^b^	1.550 ^b^	0.724 ^b^	0.032 ^a^	0.294 ^abc^	0.399 ^abc^	0.588 ^b^
SG.D2W5	0.199 ^a^	0.335 ^b^	1.093 ^b^	1.291 ^b^	0.525 ^b^	0.039 ^a^	0.318 ^ab^	0.291 ^bc^	0.330 ^b^
SG.D2W11	0.210 ^a^	0.341 ^b^	1.031 ^b^	1.215 ^b^	0.536 ^b^	0.059 ^a^	0.301 ^ab^	0.363 ^bc^	0.322 ^b^

RMSEp = Root Mean Square Error of Prediction, R^2^p = Coefficient of determination for the prediction set, RPD = Ratio of performance to deviation, RPIQ = Ratio of Performance to Interquartile distance, CCC = Concordance Correlation Coefficient, RMSEcv = Root Mean Square Error of Cross-validation, R^2^cv = Coefficient of Determination for cross-validation, R^2^sp = squared Spearman’s rank correlation coefficient. Means within the same column followed by the same superscript letter are not statistically significantly different at α = 0.05.

**Table 3:** Mean performance statistics of NIRS for predicting composition of Caffeine in green Arabica coffee beans using the SVM algorithm.

Pretreatment	RMSEp	R^2^p	RPD	RPIQ	CCC	Bias	R^2^sp
Raw_data	0.071 ^c^	0.894 ^a^	3.342 ^a^	3.992 ^a^	0.939 ^a^	0.015 ^a^	0.920 ^a^
SG	0.071 ^c^	0.895 ^a^	3.349 ^a^	4.003 ^a^	0.940 ^a^	0.015 ^a^	0.924 ^a^
SNV	0.096 ^bc^	0.806 ^a^	2.308 ^a^	2.760 ^a^	0.887 ^a^	0.014 ^a^	0.788 ^a^
SNVSG	0.097 ^bc^	0.805 ^a^	2.304 ^a^	2.756 ^a^	0.886 ^a^	0.015 ^a^	0.788 ^a^
SNV1D	0.448 ^abc^	0.185 ^b^	0.498 ^b^	0.587 ^b^	0.304 ^b^	0.011 ^a^	0.231 ^b^
SNV2D	0.437 ^abc^	0.199 ^b^	0.604 ^b^	0.692 ^b^	0.343 ^b^	0.006 ^a^	0.322 ^b^
D1	0.464 ^ab^	0.161 ^b^	0.476 ^b^	0.561 ^b^	0.276 ^b^	0.028 ^a^	0.195 ^b^
D2	0.503 ^a^	0.179 ^b^	0.537 ^b^	0.613 ^b^	0.306 ^b^	0.002 ^a^	0.300 ^b^
SGD1	0.326 ^abc^	0.332 ^b^	0.840 ^b^	0.976 ^a^	0.498 ^b^	0.007 ^a^	0.370 ^b^
SG.D1W5	0.471 ^ab^	0.155 ^b^	0.468 ^b^	0.553 ^a^	0.270 ^b^	0.022 ^a^	0.184 ^b^
SG.D1W11	0.476 ^ab^	0.139 ^b^	0.460 ^b^	0.547 ^a^	0.260 ^b^	0.003 ^a^	0.175 ^b^
SG.D2W5	0.600 ^a^	0.148 ^b^	0.480 ^b^	0.545 ^a^	0.260 ^b^	−0.018 ^a^	0.275 ^b^
SG.D2W11	0.616 ^a^	0.152 ^b^	0.473 ^b^	0.536 ^a^	0.261 ^b^	−0.025 ^a^	0.287 ^b^

RMSEp = Root Mean Square Error of Prediction, R^2^p = Coefficient of determination for the prediction set, RPD = Ratio of performance to deviation, RPIQ = Ratio of Performance to Interquartile distance, CCC = Concordance Correlation Coefficient, R^2^sp = squared Spearman’s rank correlation coefficient. Means within the same column followed by the same superscript letter are not statistically significantly different at α = 0.05.

**Table 4:** Mean performance statistics of NIRS for predicting composition of Caffeine in green Arabica coffee beans using the RF algorithm.

Pretreatment	RMSEp	R^2^p	RPD	RPIQ	CCC	Bias	R^2^sp
Raw_data	0.094 ^d^	0.835 ^c^	2.309 ^d^	2.742 ^d^	0.891 ^b^	0.03 ^a^	0.843 ^a^
SG	0.095 ^d^	0.826 ^bc^	2.312 ^d^	2.743 ^d^	0.890 ^c^	0.031 ^a^	0.831 ^a^
SNV	0.127 ^ab^	0.659 ^ab^	1.690 ^b^	2.007 ^b^	0.769 ^a^	0.015 ^a^	0.577 ^d^
SNV1D	0.125 ^b^	0.684 ^b^	1.722 ^b^	2.040 ^b^	0.779 ^a^	0.023 ^a^	0.680 ^d^
SNV2D	0.147 ^a^	0.566 ^a^	1.457 ^a^	1.727 ^a^	0.666 ^a^	0.038 ^a^	0.62 ^b^
D1	0.125 ^cd^	0.682 ^a^	1.730 ^bc^	2.062 ^bc^	0.783 ^a^	0.021 ^a^	0.632 ^b^
D2	0.151 ^c^	0.541 ^a^	1.438 ^b^	1.702 ^b^	0.654 ^a^	0.039 ^a^	0.564 ^c^
SNVSG	0.126 ^ab^	0.670 ^a^	1.712 ^a^	2.039 ^a^	0.776 ^a^	0.014 ^a^	0.597 ^d^
SGD1	0.123 ^cd^	0.689 ^cd^	1.755 ^d^	2.083 ^d^	0.785 ^b^	0.019 ^a^	0.623 ^b^
SG.D1W5	0.121 ^cd^	0.710 ^cd^	1.785 ^cd^	2.129 ^cd^	0.787 ^b^	0.019 ^a^	0.671 ^b^
SG.D1W11	0.121 ^cd^	0.701 ^cd^	1.789 ^bc^	2.132 ^bc^	0.786 ^a^	0.022 ^a^	0.651 ^b^
SG.D2W5	0.149 ^c^	0.546 ^c^	1.459 ^bc^	1.725 ^bc^	0.663 ^a^	0.034 ^a^	0.566 ^bc^
SG.D2W11	0.146 ^c^	0.561 ^c^	1.480 ^b^	1.754 ^b^	0.677 ^a^	0.035 ^a^	0.595 ^bc^

RMSEP = Root Mean Square Error of Prediction, R^2^p = Coefficient of determination for the prediction set, RPD = Ratio of performance to deviation, RPIQ = Ratio of Performance to Interquartile distance, CCC = Concordance Correlation Coefficient, R^2^sp = squared Spearman’s rank correlation coefficient. Means within the same column followed by the same superscript letter are not statistically significantly different at α = 0.05.

**Table 5:** Mean performance statistics of NIRS for predicting composition of Protein content in green Arabica coffee beans using the PLS algorithm.

Pretreatment	RMSEp	R^2^p	RPD	RPIQ	CCC	Bias	RMSEcv	R^2^cv	R^2^sp
Raw_data	0.145 ^d^	0.977 ^a^	6.915 ^a^	7.079 ^a^	0.988 ^a^	−0.010 ^a^	0.172 ^cd^	0.967 ^a^	0.962 ^a^
SG	0.144 ^d^	0.978 ^a^	6.922 ^a^	7.086 ^a^	0.988 ^a^	−0.010 ^a^	0.172 ^cd^	0.967 ^a^	0.962 ^a^
SNV	0.316 ^bc^	0.897 ^bc^	3.474 ^bcd^	3.503	0.939 ^bcd^	−0.028 ^a^	0.167 ^d^	0.968 ^a^	0.923 ^b^
SNV1D	0.258 ^c^	0.930 ^ab^	3.847 ^bc^	3.904	0.961 ^ab^	−0.034 ^a^	0.182 ^bcd^	0.963 ^ab^	0.927 ^b^
SNV2D	0.447 ^a^	0.787 ^d^	2.196 ^e^	2.238	0.878 ^e^	−0.029 ^a^	0.230 ^a^	0.938 ^c^	0.772 ^d^
D1	0.258 ^c^	0.928 ^ab^	4.090 ^b^	4.129	0.960 ^abc^	−0.033 ^a^	0.169 ^d^	0.968 ^a^	0.933 ^b^
D2	0.371 ^ab^	0.858 ^c^	2.690 ^de^	2.729	0.920 ^d^	−0.038 ^a^	0.202 ^ab^	0.952 ^b^	0.858 ^c^
SNVSG	0.332 ^bc^	0.888 ^bc^	3.351 ^bcd^	3.373	0.933 ^bcd^	−0.037 ^a^	0.179 ^bcd^	0.963 ^ab^	0.919 ^b^
SGD1	0.259 ^c^	0.928 ^ab^	4.019 ^b^	4.051	0.960 ^abc^	−0.028 ^a^	0.170 ^d^	0.967 ^a^	0.929 ^b^
SG.D1W5	0.256 ^c^	0.929 ^ab^	4.124 ^b^	4.162	0.960 ^abc^	−0.031 ^a^	0.168 ^d^	0.968 ^a^	0.936 ^b^
SG.D1W11	0.253 ^c^	0.931 ^ab^	4.151 ^b^	4.191	0.961 ^abc^	−0.031 ^a^	0.169 ^d^	0.968 ^a^	0.932 ^b^
SG.D2W5	0.368 ^ab^	0.860 ^c^	2.714 ^de^	2.753	0.921 ^d^	−0.040 ^a^	0.200 ^ab^	0.953 ^b^	0.860 ^c^
SG.D2W11	0.353 ^ab^	0.870 ^bc^	2.859 ^de^	2.894	0.927 ^d^	−0.045 ^a^	0.203 ^ab^	0.953 ^b^	0.875 ^c^

RMSEp = Root Mean Square Error of Prediction, R^2^p = Coefficient of determination for the prediction set, RPD = Ratio of performance to deviation, RPIQ = Ratio of Performance to Interquartile distance, CCC = Concordance Correlation Coefficient, RMSEcv = Root Mean Square Error of Cross-validation, R^2^cv = Coefficient of Determination for cross-validation, R^2^sp = squared Spearman’s rank correlation coefficient. Means within the same column followed by the same superscript letter are not statistically significantly different at α = 0.05.

**Table 6:** Mean performance statistics of NIRS for predicting composition of Protein content in green Arabica coffee beans using the SVM algorithm.

Pretreatment	RMSEp	R^2^p	RPD	RPIQ	CCC	Bias	R^2^sp
Raw_data	0.174 ^d^	0.968 ^a^	5.772 ^a^	5.870 ^a^	0.982 ^a^	−0.016 ^a^	0.953 ^a^
SG	0.174 ^d^	0.968 ^a^	5.772 ^a^	5.869 ^a^	0.982 ^a^	−0.017 ^a^	0.952 ^a^
SNV	0.246 ^cd^	0.933 ^ab^	4.322 ^b^	4.357 ^b^	0.962 ^ab^	−0.029 ^a^	0.933 ^a^
SNV1D	0.317 ^abc^	0.901 ^bcde^	3.218 ^c^	3.243 ^cd^	0.942 ^bcd^	−0.048 ^a^	0.898 ^b^
SNV2D	0.398 ^a^	0.827 ^f^	2.559 ^c^	2.580 ^d^	0.902 ^e^	−0.014 ^a^	0.855 ^c^
D1	0.294 ^ab^	0.913 ^abc^	3.389 ^bc^	3.425 ^c^	0.951 ^abc^	−0.047 ^a^	0.900 ^b^
D2	0.367 ^ab^	0.858 ^cdef^	2.938 ^c^	2.963 ^cd^	0.920 ^cde^	−0.013 ^a^	0.885 ^b^
SNVSG	0.245 ^cd^	0.934 ^ab^	4.325 ^b^	4.361 ^b^	0.963 ^ab^	−0.029 ^a^	0.933 ^a^
SGD1	0.367 ^ab^	0.868 ^cdef^	2.942 ^c^	2.965 ^cd^	0.922 ^cde^	−0.053 ^a^	0.880 ^bc^
SG.D1W5	0.294 ^ab^	0.912 ^abc^	3.375 ^bc^	3.412 ^c^	0.951 ^abc^	−0.046 ^a^	0.899 ^b^
SG.D1W11	0.300 ^ab^	0.909 ^def^	3.302 ^c^	3.341 ^cd^	0.949 ^cd^	−0.043 ^a^	0.897 ^b^
SG.D2W5	0.383 ^ab^	0.847 ^ef^	2.865 ^c^	2.886 ^cd^	0.913 ^de^	−0.011 ^a^	0.882 ^bc^
SG.D2W11	0.379 ^ab^	0.851 ^def^	2.899 ^c^	2.918 ^cd^	0.915 ^cde^	−0.010 ^a^	0.884 ^b^

RMSEp = Root Mean Square Error of Prediction, R^2^p = Coefficient of determination for the prediction set, RPD = Ratio of performance to deviation, RPIQ = Ratio of Performance to Interquartile distance, CCC = Concordance Correlation Coefficient, R^2^sp = squared Spearman’s rank correlation coefficient. Means within the same column followed by the same superscript letter are not statistically significantly different at α = 0.05.

**Table 7:** Mean performance statistics of NIRS for predicting composition of Protein content in green Arabica coffee beans using the RF algorithm.

Pretreatment	RMSEp	R^2^p	RPD	RPIQ	CCC	Bias	R^2^sp
Raw_data	0.180 ^d^	0.966 ^a^	5.404 ^a^	5.536 ^a^	0.981 ^a^	−0.004 ^a^	0.955 ^a^
SG	0.181 ^d^	0.965 ^a^	5.393 ^a^	5.521 ^a^	0.981 ^a^	−0.004 ^a^	0.954 ^a^
SNV	0.407 ^b^	0.825 ^c^	2.434 ^d^	2.487 ^d^	0.900 ^c^	−0.006 ^a^	0.828 ^d^
SNV1D	0.373 ^b^	0.855 ^c^	2.589 ^d^	2.652 ^d^	0.906 ^c^	−0.007 ^a^	0.832 ^d^
SNV2D	0.459 ^a^	0.780 ^d^	2.107 ^d^	2.166 ^d^	0.848 ^d^	−0.008 ^a^	0.723 ^e^
D1	0.235 ^c^	0.941 ^ab^	4.234 ^b^	4.317 ^b^	0.965 ^ab^	−0.016 ^a^	0.917 ^bc^
D2	0.276 ^c^	0.920 ^b^	3.545 ^c^	3.630 ^c^	0.951 ^b^	−0.014 ^a^	0.890 ^c^
SNVSG	0.408 ^b^	0.825 ^c^	2.436 ^d^	2.488 ^d^	0.900 ^c^	−0.010 ^a^	0.828 ^d^
SGD1	0.231 ^c^	0.943 ^ab^	4.303 ^b^	4.387 ^b^	0.967 ^ab^	−0.017 ^a^	0.919 ^b^
SG.D1W5	0.233 ^c^	0.942 ^ab^	4.258 ^b^	4.340 ^b^	0.966 ^ab^	−0.017 ^a^	0.918 ^b^
SG.D1W11	0.233 ^c^	0.941 ^ab^	4.259 ^b^	4.340 ^b^	0.966 ^ab^	−0.015 ^a^	0.917 ^bc^
SG.D2W5	0.272 ^c^	0.923 ^b^	3.596 ^c^	3.677 ^c^	0.953 ^b^	−0.010 ^a^	0.892 ^bc^
SG.D2W11	0.275 ^c^	0.920 ^b^	3.562 ^c^	3.642 ^c^	0.952 ^b^	−0.012 ^a^	0.893 ^bc^

RMSEp = Root Mean Square Error of Prediction, R^2^p = Coefficient of determination for the prediction set, RPD = Ratio of performance to deviation, RPIQ = Ratio of Performance to Interquartile distance, CCC = Concordance Correlation Coefficient, R^2^sp = squared Spearman’s rank correlation coefficient. Means within the same column followed by the same superscript letter are not statistically significantly different at α = 0.05.

**Table 8:** Mean performance statistics of NIRS for predicting composition of Trigonelline content in green Arabica coffee beans using the PLS algorithm.

Pretreatment	RMSEp	R^2^p	RPD	CCC	RPIQ	Bias	RMSEcv	R^2^cv	R^2^sp
Raw_data	0.007 ^f^	0.990 ^a^	10.528 ^a^	0.994 ^a^	12.469 ^a^	0.001 ^a^	0.010 ^e^	0.978 ^a^	0.982 ^a^
SG	0.007 ^f^	0.990 ^a^	10.533 ^a^	0.994 ^a^	12.476 ^a^	0.001 ^a^	0.010 ^e^	0.978 ^a^	0.982 ^a^
SNV	0.031 ^abc^	0.796 ^cd^	2.329 ^b^	0.875 ^cd^	2.768 ^b^	−0.001 ^a^	0.030 ^a^	0.779 ^e^	0.775 ^ef^
SNV1D	0.020 ^de^	0.910 ^ab^	3.495 ^b^	0.946 ^ab^	4.153 ^b^	0.001 ^a^	0.021 ^cd^	0.892 ^bc^	0.918 ^bc^
SNV2D	0.034 ^da^	0.762 ^d^	2.079 ^b^	0.843 ^d^	2.475 ^b^	0.002 ^a^	0.025 ^b^	0.853 ^cd^	0.734 ^f^
D1	0.020 ^de^	0.913 ^ab^	3.537 ^b^	0.946 ^ab^	4.202 ^b^	0.002 ^a^	0.021 ^cd^	0.890 ^bc^	0.928 ^ab^
D2	0.027 ^abcd^	0.845 ^bcd^	2.498 ^b^	0.901 ^bc^	2.964 ^b^	0.001 ^a^	0.023 ^bc^	0.870 ^cd^	0.808 ^e^
SNVSG	0.031 ^ab^	0.794 ^cd^	2.314 ^b^	0.873 ^cd^	2.750 ^b^	−0.001 ^a^	0.030 ^a^	0.777 ^e^	0.775 ^ef^
SGD1	0.020 ^e^	0.918 ^ab^	3.574 ^b^	0.948 ^ab^	4.247 ^b^	0.002 ^a^	0.021 ^cd^	0.888 ^bc^	0.932 ^ab^
SG.D1W5	0.021 ^de^	0.912 ^ab^	3.524 ^b^	0.946 ^ab^	4.185 ^b^	0.002 ^a^	0.021 ^cd^	0.891 ^bc^	0.926 ^ab^
SG.D1W11	0.020 ^e^	0.919 ^ab^	3.659 ^b^	0.950 ^ab^	4.346 ^b^	0.002 ^a^	0.021 ^cd^	0.893 ^bc^	0.933 ^ab^
SG.D2W5	0.026 ^bcde^	0.859 ^bc^	2.618 ^b^	0.909 ^bc^	3.106 ^b^	0.001 ^a^	0.022 ^cd^	0.881 ^bcd^	0.829 ^de^
SG.D2W11	0.024 ^cde^	0.883 ^bc^	2.883 ^b^	0.926 ^b^	3.421 ^b^	0.002 ^a^	0.020 ^d^	0.902 ^b^	0.867 ^cd^

RMSEp = Root Mean Square Error of Prediction, R^2^p = Coefficient of determination for the prediction set, RPD = Ratio of performance to deviation, RPIQ = Ratio of Performance to Interquartile distance, CCC = Concordance Correlation Coefficient, RMSEcv = Root Mean Square Error of Cross-validation, R^2^cv = Coefficient of Determination for cross-validation, R^2^sp = squared Spearman’s rank correlation coefficient. Means within the same column followed by the same superscript letter are not statistically significantly different at α = 0.05.

**Table 9:** Mean performance statistics of NIRS for predicting composition of Trigonelline content in green Arabica coffee beans using the SVM algorithm (Table 8).

Pretreatment	RMSEp	R^2^p	RPD	CCC	Bias	R^2^sp
Raw_data	0.008 ^d^	0.968 ^a^	9.110 ^a^	0.993 ^a^	0.001 ^a^	0.969 ^a^
SG	0.008 ^d^	0.968 ^a^	9.117 ^a^	0.993 ^a^	0.001 ^a^	0.970 ^a^
SNV	0.013 ^c^	0.965 ^ab^	5.337 ^b^	0.981 ^ab^	0.001 ^a^	0.941 ^ab^
SNV1D	0.017 ^bc^	0.935 ^b^	4.095 ^bc^	0.965 ^b^	0.001 ^a^	0.913 ^bc^
SNV2D	0.022 ^a^	0.902 ^c^	3.167 ^bc^	0.937 ^d^	0.002 ^a^	0.890 ^d^
D1	0.017 ^c^	0.938 ^b^	4.221 ^bc^	0.967 ^b^	0.001 ^a^	0.916 ^abc^
D2	0.019 ^a^	0.921 ^c^	3.517 ^bc^	0.954 ^cd^	0.001 ^a^	0.903 ^cd^
SNVSG	0.013 ^c^	0.966 ^ab^	5.379 ^bc^	0.982 ^ab^	0.001 ^a^	0.939 ^abc^
SGD1	0.022 ^bc^	0.896 ^b^	3.245 ^bc^	0.944 ^b^	0.002 ^a^	0.863 ^bc^
SG.D1W5	0.018 ^c^	0.933 ^b^	4.122 ^bc^	0.964 ^b^	0.001 ^a^	0.913 ^abc^
SG.D1W11	0.018 ^a^	0.930 ^c^	4.001 ^bc^	0.962 ^cd^	0.002 ^a^	0.910 ^bcd^
SG.D2W5	0.020 ^ab^	0.914 ^c^	3.440 ^bc^	0.952 ^c^	0.000 ^a^	0.899 ^bc^
SG.D2W11	0.020 ^c^	0.918 ^b^	3.540 ^bc^	0.954 ^b^	0.001 ^a^	0.906 ^bc^

RMSEp = Root Mean Square Error of Prediction, R^2^p = Coefficient of determination for the prediction set, RPD = Ratio of performance to deviation, RPIQ = Ratio of Performance to Interquartile distance, CCC = Concordance Correlation Coefficient, R^2^sp = squared Spearman’s rank correlation coefficient. Means within the same column followed by the same superscript letter are not statistically significantly different at α = 0.05.

**Table 10:** Mean performance statistics of NIRS for predicting composition of Trigonelline content in green Arabica coffee beans using the RF algorithm.

Pretreatment	RMSEp	R^2^p	RPD	RPIQ	CCC	Bias	R^2^sp
Raw_data	0.009 ^f^	0.958 ^a^	8.528 ^a^	6.204 ^a^	0.991 ^a^	0.001 ^a^	0.975 ^a^
SG	0.008 ^f^	0.958 ^a^	8.619 ^a^	6.227 ^a^	0.991 ^a^	0.001 ^a^	0.976 ^a^
SNV	0.022 ^cd^	0.907 ^c^	3.135 ^cde^	3.108 ^cd^	0.938 ^de^	−0.001 ^a^	0.873 ^cd^
SNV1D	0.027 ^b^	0.857 ^d^	2.491 ^ef^	2.590 ^de^	0.894 ^f^	0.002 ^a^	0.844 ^d^
SNV2D	0.036 ^a^	0.739 ^e^	1.901 ^f^	2.120 ^e^	0.793 ^g^	0.003 ^a^	0.708 ^e^
D1	0.019 ^e^	0.930 ^b^	3.690 ^bcd^	3.731 ^b^	0.956 ^bc^	0.001 ^a^	0.909 ^ab^
D2	0.023 ^de^	0.891 ^bc^	2.923 ^bcd^	3.399 ^bc^	0.928 ^d^	0.001 ^a^	0.876 ^bc^
SNVSG	0.022 ^c^	0.909 ^c^	3.140 ^de^	3.084 ^cd^	0.938 ^e^	0.000 ^a^	0.874 ^cd^
SGD1	0.018 ^e^	0.932 ^b^	3.783 ^b^	3.788 ^b^	0.957 ^b^	0.001 ^a^	0.907 ^ab^
SG.D1W5	0.019 ^e^	0.930 ^b^	3.686 ^bc^	3.750 ^b^	0.956 ^bc^	0.001 ^a^	0.906 ^ab^
SG.D1W11	0.019 ^e^	0.930 ^b^	3.737 ^bc^	3.751 ^b^	0.956 ^b^	0.001 ^a^	0.906 ^ab^
SG.D2W5	0.023 ^de^	0.899 ^bc^	2.969 ^bcd^	3.406 ^bc^	0.930 ^cd^	0.001 ^a^	0.878 ^bc^
SG.D2W11	0.022 ^cde^	0.908 ^bc^	3.041 ^bcd^	3.374 ^bc^	0.933 ^de^	0.001 ^a^	0.889 ^bc^

RMSEp = Root Mean Square Error of Prediction, R^2^p = Coefficient of determination for the prediction set, RPD = Ratio of performance to deviation, RPIQ = Ratio of Performance to Interquartile distance, CCC = Concordance Correlation Coefficient, R^2^sp = squared Spearman’s rank correlation coefficient. Means within the same column followed by the same superscript letter are not statistically significantly different at α = 0.05.

**Table 11:** HPLC parameters set using a reverse-phase C18 column, a water–organic solvent mobile phase, 10 µL injection volume, 30 °C column temperature, 1.0 mL min^−1^ flow rate, and UV detection at 273 nm.

Parameter Requested	Status or Quantity
Injection volume	10 µL
Column temperature	25–30 °C
Column type/dimensions	Reverse-phase C18 column, commonly 250 × 4.6 mm, 5 µm or 150 × 4.6 mm, 5 µm
Mobile phase composition	Water:acetonitrile, commonly 80:20 or 70:30 *v*/*v*; may include 0.1% formic/acetic acid
Flow rate	0.8–1.0 mL/min; commonly 1.0 mL/min
Run time/acquisition method	5–15 min per sample; caffeine peak often around 3–8 min, depending on column/mobile phase
Detector settings beyond MS transition	UV/PDA detection commonly at 272–280 nm; caffeine monitored at 273 nm

## Data Availability

The raw data supporting the conclusions of this article will be made available by the authors on request. The data are not publicly available due to institutional policies and restrictions.

## Supplementary Materials

The following supporting information can be downloaded at: https://www.mdpi.com/article/10.3390/plants15142117/s1. Table S1: Summary statistics of the models across 10 iterations; Table S2: Data Quality parameters for HPLC generated reference data; Table S3: Spectral Pretreatment Methods and Their Roles in Correcting Spectral Defects Improved Prediction Accuracy [34,48,80,81]; Figure S1: HPLC Chromatograms for caffeine content determination.
