# Supplementary material for: Rapid Analysis of Caffeine, Protein and Trigonelline in Ugandan Arabica Coffee Using NIRS and Machine Learning Algorithms
Source: Plants (Basel). 2026 Jul 9;15(14):2117. doi: 10.3390/plants15142117 (PMC13416277; doi:10.3390/plants15142117)
Supplement: Supplementary file 1 [file plants-15-02117-s001.zip › Supplementary Table S3.pdf]

Supplementary Table S3: Spectral Pretreatment Methods and Their Roles in Correcting Spectral Defects Improved Prediction Accuracy

Pretreatment	Code	Role in Correcting Spectral Defects	Reference
Raw data (untreated spectra)	1	Baseline reference; no correction applied. Included to evaluate the effect of all other pretreatments against unprocessed spectra.	
Standard Normal Variate (SNV)	2	Corrects multiplicative effects caused by light scatter, particle size variations, and path length differences by centering and scaling each spectrum row-wise. Normalizes spectral data to remove physical scattering artifacts.	[1]
SNV and first derivative	3	Combines scatter correction (SNV) with first derivative to remove baseline offsets and enhance spectral features. The derivative isolates chemical information by removing additive effects.	[1]
SNV and second derivative	4	Combines scatter correction (SNV) with second derivative to correct for both multiplicative scatter and non-linear baseline curvature while enhancing resolution of overlapping peaks.	[1]
First derivative	5	Removes constant baseline offsets and additive noise by calculating the rate of change (slope) of the spectrum. Enhances sharp spectral features and improves resolution of overlapping bands.	[2]
Second derivative	6	Removes linear baseline trends and corrects for baseline curvature. Provides enhanced resolution of severely overlapping peaks compared to first derivative.	[3]
Savitzky–Golay filter (SG)	7	Reduces high-frequency random noise, measurement artifacts, and electronic fluctuations while preserving spectral features.	[4]
SNV and Savitzky–Golay filter	8	Two-step correction: SNV removes scatter effects (physical noise), followed by SG smoothing to reduce high-frequency random noise. Provides comprehensive noise reduction addressing both multiplicative and additive noise sources.	[2,4]
Gap-segment derivative (window size = 11)	9	Calculates derivative using a gap between segments to reduce the influence of local noise and baseline variations.	

Pretreatment	Code	Role in Correcting Spectral Defects	Reference
Savitzky–Golay and first derivative (window size = 5)	10	Combines SG smoothing with first derivative using a small window (5 points). Smoothing reduces random noise before derivative calculation, which removes baseline offsets. The small window preserves sharp spectral features for high-resolution analysis.	[4]
Savitzky–Golay and first derivative (window size = 11)	11	Same as Savitzky–Golay and first derivative (window size = 5) but with a larger window (11 points).	[3,4]
Savitzky–Golay and second derivative (window size = 5)	12	Combines SG smoothing with second derivative using a small window (5 points). Removes baseline curvature and resolves overlapping peaks while smoothing reduces high-frequency noise.	[2,4]
Savitzky–Golay and second derivative (window size = 11)	13	Same as Savitzky–Golay and second derivative (window size = 5) but with a larger window (11 points).	[2,4]

References

[1] Barnes RJ, Dhanoa MS, Lister SJ. Standard normal variate transformation and de-trending of near-infrared diffuse reflectance spectra. Appl Spectrosc 1989;43:772–7. https://doi.org/10.1366/0003702894202201.

[2] Shenk JS, Westerhaus MO. Populations Structuring of Near Infrared Spectra and Modified Partial Least Squares Regression. Crop Sci 1991;31:1548–55. https://doi.org/10.2135/CROPSCI1991.0011183X003100060034X.

[3] Candolfi A, De Maesschalck R, Massart DL, Hailey PA, Harrington ACE. Identification of pharmaceutical excipients using NIR spectroscopy and SIMCA. J Pharm Biomed Anal 1999;19:923–35. https://doi.org/10.1016/S0731-7085(98)00234-9.

[4] Chen H, Song Q, Tang G, Feng Q, Lin L. The Combined Optimization of Savitzky-Golay Smoothing and Multiplicative Scatter Correction for FT-NIR PLS Models. ISRN Spectroscopy 2013;2013:1–9. https://doi.org/10.1155/2013/642190.
