# Supplementary material for: Rapid Analysis of Caffeine, Protein and Trigonelline in Ugandan Arabica Coffee Using NIRS and Machine Learning Algorithms
Source: Plants (Basel). 2026 Jul 9;15(14):2117. doi: 10.3390/plants15142117 (PMC13416277; doi:10.3390/plants15142117)
Supplement: Supplementary file 1 [file plants-15-02117-s001.zip › Supplimentary Table S2.pdf]

Table S2. Data Quality parameters for HPLC generated reference data

Validation Parameter	Value/Observation	Evidence
Calibration linearity	Linear calibration model with R^2 = 0.9999985 (correlation coefficient R = 0.9999992)	0.999998
Calibration equation	y = 13715.13x − 80415.65	
Calibration range	Standards 15, 250, and 500 µg/mL (ppb standards as labelled)	
Accuracy of calibration standards	500 ppb: 100.03%; 250 ppb: 99.86%; 15.485 ppb: 101.16%	0.06%
Repeatability	Mean peak area was 3344.863 ppm with a %RSD of 0.06%	0.18%
Reproducibility (Inter-day)	%Reproducibility of measured caffeine concentration was 0.18%	99.30%
Recovery	Recovery ranged from 98.4% to 101.3%	±1.10%
Precision	0.85	0.85 µg/mL
