# Supplementary material for: Rapid Analysis of Caffeine, Protein and Trigonelline in Ugandan Arabica Coffee Using NIRS and Machine Learning Algorithms
Source: Plants (Basel). 2026 Jul 9;15(14):2117. doi: 10.3390/plants15142117 (PMC13416277; doi:10.3390/plants15142117)

Results File: E:\LCMS results_2026\evans\Caffeine\EU_Caffeine.iproc - Caffeine_Optimized_5min.lcm

S1-Western

Conc. 332.08652
Area 4474193
Q 195.10>138.10 (+) 5.91e5

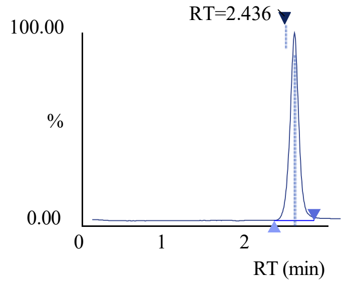

S2-Western

Conc. 336.81762
Area 4539080
Q 195.10>138.10 (+) 5.62e5

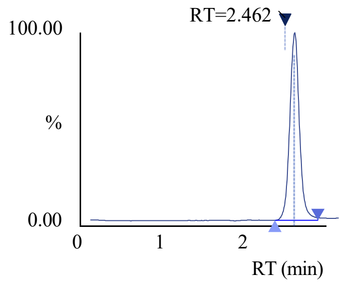

S3-Western

Conc. 377.15592
Area 5092325
Q 195.10>138.10 (+) 7.20e5

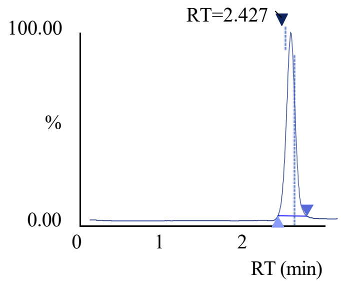

S4-Western

Conc. 388.80445
Area 5252086
Q 195.10>138.10 (+) 7.02e5

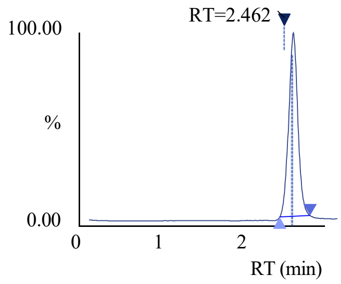

S1-Eastern

Conc. 360.02172
Area 4857328
Q 195.10>138.10 (+) 7.08e5

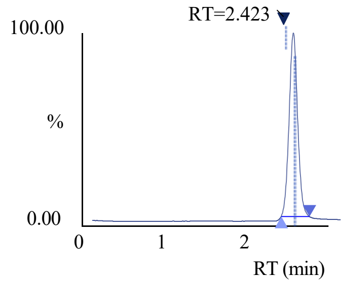

S2-Eastern

Conc. 366.07717
Area 4940379
Q 195.10>138.10 (+) 6.51e5

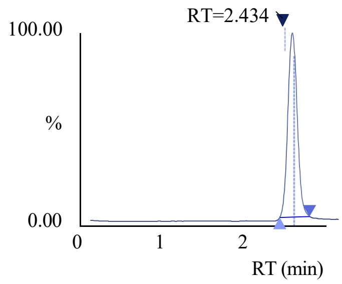

S3-Eastern

Conc. 325.74283
Area 4387188
Q 195.10>138.10 (+) 6.25e5

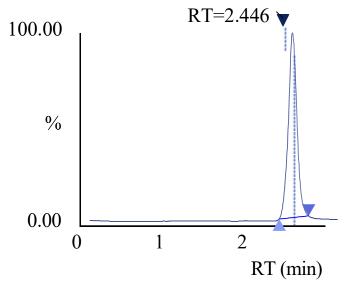

S4-Eastern

Conc. 326.18304
Area 4393226
Q 195.10>138.10 (+) 5.92e5

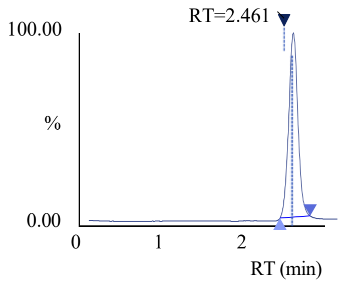

S5-Eastern

Conc. 324.70226
Area 4372917
Q 195.10>138.10 (+) 6.31e5

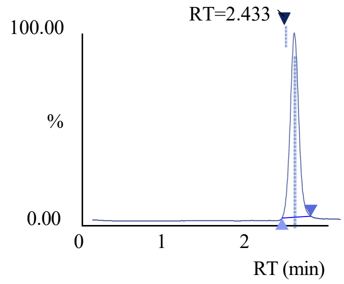

S6-Eastern

Conc. 327.00450
Area 4404492
Q 195.10>138.10 (+) 6.13e5

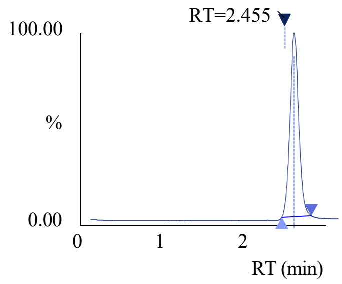

S7-Eastern

Conc. 378.03346
Area 5104361
Q 195.10>138.10 (+) 6.18e5

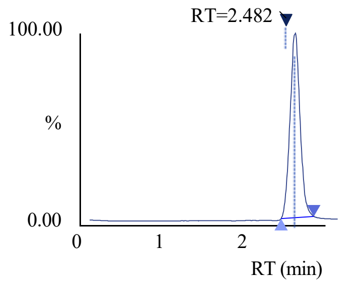

S8-Eastern

Conc. 351.38967
Area 4738938
Q 195.10>138.10 (+) 6.51e5

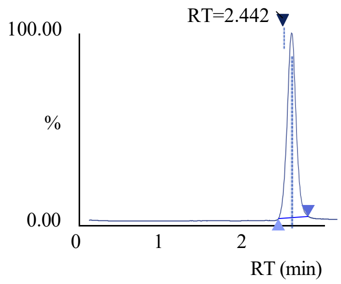

Compound: caffeine (continued)

S5-Western

Conc. 384.72517
Area 5196138
Q 195.10>138.10 (+) 6.86e5

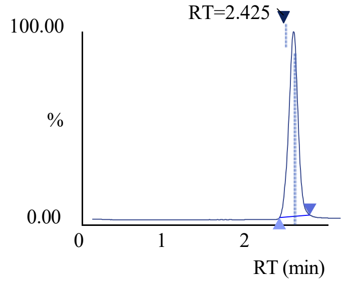

S6-Western

Conc. 377.08685
Area 5091378
Q 195.10>138.10 (+) 6.86e5

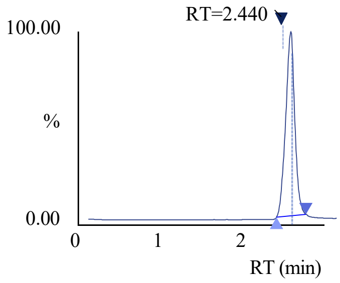

S7-western

Conc. 268.78016
Area 3605938
Q 195.10>138.10 (+) 5.17e5

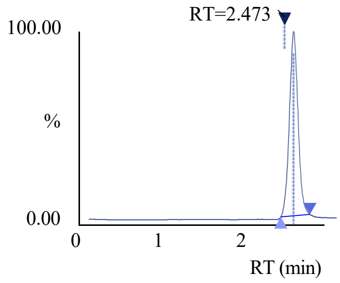

S8-Western

Conc. 261.95726
Area 3512361
Q 195.10>138.10 (+) 5.47e5

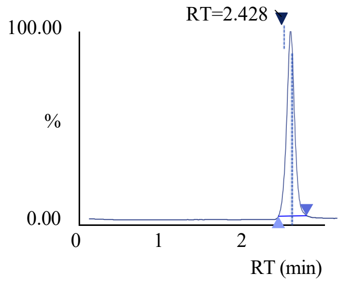

Supplement: Supplementary file 1 [file plants-15-02117-s001.zip › Supplimentatry Figure S1.HPLC Chromatograms.pdf]
